# Supplementary material for: SFPFusion: An Improved Vision Transformer Combining Super Feature Attention and Wavelet-Guided Pooling for Infrared and Visible Images Fusion
Source: Sensors (Basel). 2023 Sep 13;23(18):7870. doi: 10.3390/s23187870 (PMC10536945; doi:10.3390/s23187870)
Supplement: Supplementary file 1 [file sensors-23-07870-s001.zip › sensors-2578388-supplementary.pdf]

sensors

MDPI

Article

SFPFusion: An Improved Vision Transformer Combining Super Feature Attention and Wavelet-Guided Pooling for Infrared and Visible Images Fusion

Hui Li *,†, Yongbiao Xiao †, Chunyang Cheng and Xiaoning Song

International Joint Laboratory on Artificial Intelligence of Jiangsu Province, School of Artificial Intelligence and Computer Science, Jiangnan University, Wuxi 214122, China; yongbiao_xiao_jnu@163.com (Y.X.); chunyang_cheng@163.com (C.C.); x.song@jiangnan.edu.cn (X.S.)
* Correspondence: lihui.cv@jiangnan.edu.cn
† These authors contributed equally to this work and should be considered co-first authors.

S1. Super Feature Attention

Therefore, we iteratively run the algorithm, divided into the following steps:

At iteration i, We extract the 3×3 surrounding super features $\bar{S} \in \mathbb{R}^{mn \times C \times 9}$ corresponding to each token via unroll function. We limit the association computations from each token to only 9 surrounding super features, which speeds up the running process and reduces the amount of computation. Next, we need to compute sparse associations Q^i. In this work, we apply a strategy similar to attention guidance to compute it and the formula is defined as:

$$Q^i = \text{Softmax}\left(\frac{TS^{i-1^{\mathrm{T}}}}{\sqrt{C}}\right) \tag{S1}$$

Where C is the number of channel.

Moreover, we continuously update $\bar{S}$ and combine the surrounding tokens to S via the roll function to prepare for association normalization. To be specific, super features are updated as a weighted sum of tokens, defined as follows:

$$S^i = \left(\text{Norm}(Q^i)\right)^{\mathrm{T}} T \tag{S2}$$

Where $\text{Norm}(\cdot)$ stands for normalization operation. At this point, we can capture the super features.

Since super features mainly focus on local features of visual content, we need to apply multi-head self-attention (MHSA) to pay more attention to global long-range dependencies. The attention mechanism can be defined as:

$$\text{Attention}(\mathbf{Q}, \mathbf{K}, \mathbf{V}) = \text{softmax}\left(\frac{\mathbf{Q}\mathbf{K}^T}{\sqrt{C}}\right)\mathbf{V} \tag{S3}$$

Our use of multi-head self-attention mechanism can consider different attention distributions, so that the network can obtain meaningful information from different perspectives. We apply the MHSA to the super features $S \in \mathbb{R}^{C \times m \times n}$. Thus, the full process of MHSA for S is formulated as:

$$\begin{aligned} \text{Attention}(S) &= \text{softmax}\left(\frac{\mathbf{Q}(S)\mathbf{K}^T(S)}{\sqrt{C}}\right)\mathbf{V}(S) \\ s.t. \quad \{\mathbf{Q}, \mathbf{K}, \mathbf{V}\} &= \left\{S\mathbf{W}^Q, S\mathbf{W}^K, S\mathbf{W}^V\right\} \end{aligned} \tag{S4}$$

check for updates

Citation: Li, H.; Xiao, Y.; Cheng, C.; Song, X. SFPFusion: An Improved Vision Transformer Combining Super Feature Attention and Wavelet-Guided Pooling for Infrared and Visible Images Fusion. *Sensors* **2023**, *23*, 7870. https://doi.org/10.3390/s23187870

Academic Editors: Guanqiu Qi, Yu Liu, Zhiqin Zhu and Huafeng Li

Received: 10 August 2023
Revised: 8 September 2023
Accepted: 11 September 2023
Published: 13 September 2023

Copyright: © 2023 by the authors. Licensee MDPI, Basel, Switzerland. This article is an open access article distributed under the terms and conditions of the Creative Commons Attribution (CC BY) license (https://creativecommons.org/licenses/by/4.0/).

Although super feature can capture global features well through multi-head self-attention, it also loses local details in the sampling process. So it is necessary to map super features back to the token space. Specifically, we perform the sparse multiplication of Q and S via unroll operation to upsample tokens, which can be formulated as:

$$F_{SF} = \mathrm{Up}(\mathrm{Attention}(S)) = Q\,\mathrm{Attention}(S) \tag{S5}$$

Where $\mathrm{Up}(\cdot)$ represents upsample operation.

S2. Comparative Experiments

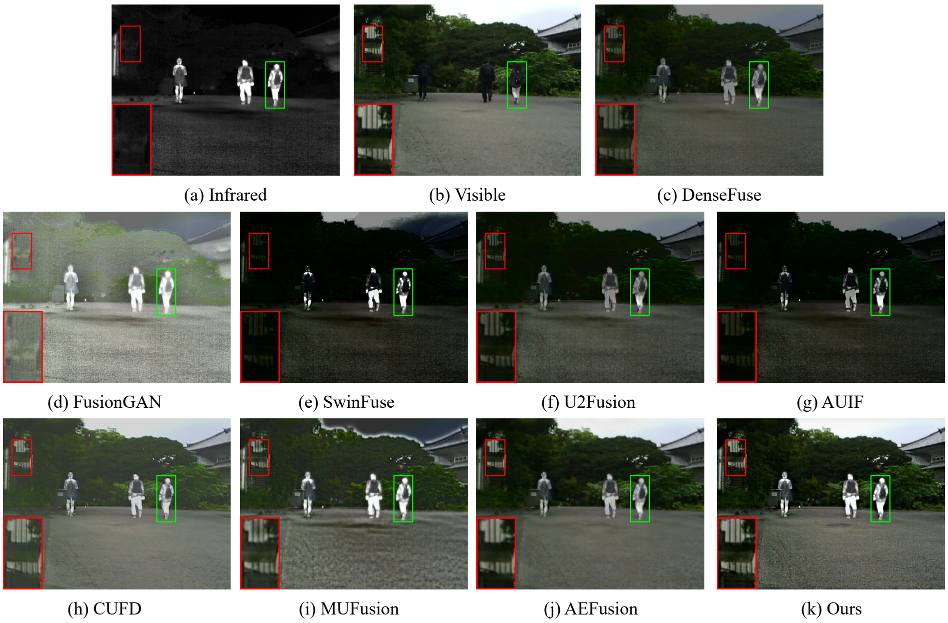

Figure S1. Qualitative comparison of our method with 8 state-of-the-art methods on the MSRS dataset.

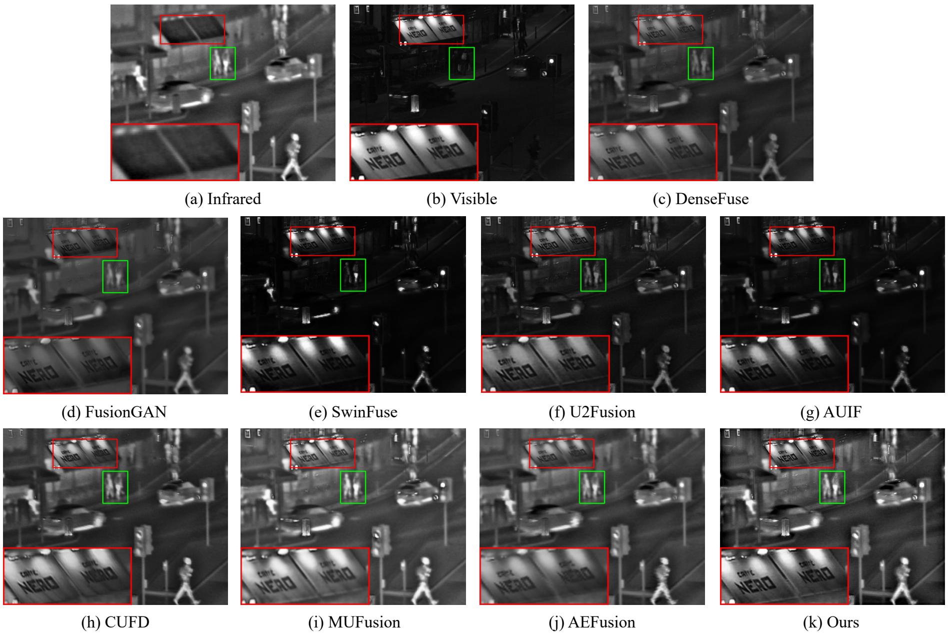

Figure S2. Qualitative comparison of our method with 8 state-of-the-art methods on the TNO dataset.
